# Efficient and Scalable Purification of Cardiomyocytes from Human Embryonic and Induced Pluripotent Stem Cells by VCAM1 Surface Expression

**DOI:** 10.1371/journal.pone.0023657

**Published:** 2011-08-18

**Authors:** Hideki Uosaki, Hiroyuki Fukushima, Ayako Takeuchi, Satoshi Matsuoka, Norio Nakatsuji, Shinya Yamanaka, Jun K. Yamashita

**Affiliations:** 1 Laboratory of Stem Cell Differentiation, Stem Cell Research Center, Institute for Frontier Medical Sciences, Kyoto University, Kyoto, Japan; 2 Department of Cell Growth and Differentiation, Center for iPS Cell Research and Application, Kyoto University, Kyoto, Japan; 3 Department of Physiology and Biophysics, Kyoto University Graduate School of Medicine, Kyoto, Japan; 4 Center for Innovation in Immunoregulative Technology and Therapeutics, Kyoto University Graduate School of Medicine, Kyoto, Japan; 5 Department of Development and Differentiation, Institute for Frontier Medical Sciences, Kyoto University, Kyoto, Japan; 6 Department of Reprogramming Science, Center for iPS Cell Research and Application, Kyoto University, Kyoto, Japan; Clinica Universidad de Navarra, Spain

## Abstract

**Rationale:**

Human embryonic and induced pluripotent stem cells (hESCs/hiPSCs) are promising cell sources for cardiac regenerative medicine. To realize hESC/hiPSC-based cardiac cell therapy, efficient induction, purification, and transplantation methods for cardiomyocytes are required. Though marker gene transduction or fluorescent-based purification methods have been reported, fast, efficient and scalable purification methods with no genetic modification are essential for clinical purpose but have not yet been established. In this study, we attempted to identify cell surface markers for cardiomyocytes derived from hESC/hiPSCs.

**Method and Result:**

We adopted a previously reported differentiation protocol for hESCs based on high density monolayer culture to hiPSCs with some modification. Cardiac troponin-T (TNNT2)-positive cardiomyocytes appeared robustly with 30–70% efficiency. Using this differentiation method, we screened 242 antibodies for human cell surface molecules to isolate cardiomyocytes derived from hiPSCs and identified anti-VCAM1 (Vascular cell adhesion molecule 1) antibody specifically marked cardiomyocytes. TNNT2-positive cells were detected at day 7–8 after induction and 80% of them became VCAM1-positive by day 11. Approximately 95–98% of VCAM1-positive cells at day 11 were positive for TNNT2. VCAM1 was exclusive with CD144 (endothelium), CD140b (pericytes) and TRA-1-60 (undifferentiated hESCs/hiPSCs). 95% of MACS-purified cells were positive for TNNT2. MACS purification yielded 5−10×10^5^ VCAM1-positive cells from a single well of a six-well culture plate. Purified VCAM1-positive cells displayed molecular and functional features of cardiomyocytes. VCAM1 also specifically marked cardiomyocytes derived from other hESC or hiPSC lines.

**Conclusion:**

We succeeded in efficiently inducing cardiomyocytes from hESCs/hiPSCs and identifying VCAM1 as a potent cell surface marker for robust, efficient and scalable purification of cardiomyocytes from hESC/hiPSCs. These findings would offer a valuable technological basis for hESC/hiPSC-based cell therapy.

## Introduction

Recent advances of stem cell biology have provided a basis of novel regenerative therapy, in which human embryonic stem cells (hESCs) and induced pluripotent stem cells (hiPSCs) can provide cardiomyocytes for transplantation [1]. To establish hESC/hiPSC-based cardiac cell therapy, efficient induction, purification and transplantation methods for cardiomyocytes are required. High differentiation efficiencies of cardiomyocytes (approximately 30–80%) have been reported in some protocols [1]–[3]. Nevertheless, these efficient methods still did not provide pure cardiomyocytes. Contamination of undifferentiated hESC/hiPSCs would cause teratoma formation after transplantation. Moreover, for application of hESC/hiPSC-derived cardiomyocytes to clinical purpose, large-scale purification with no genetic modification would be required. Thus, the establishment of human cardiomyocyte purification methods with cell surface markers has been long awaited.

We have been investigating cardiovascular cell differentiation and regeneration using mouse and human ESCs and iPSCs. We reported a systematic cardiovascular cell differentiation method with mouse iPSCs [4] and an enhancement method of hiPSC differentiation to cardiomyocytes with an immunosuppressant, cyclosporin-A [5]. In this study, to further improve differentiation efficiency of hiPSCs to cardiomyocytes and identify cell surface markers for human cardiomyocytes, we adopted an efficient differentiation method that was previously established in hESCs [1] to hiPSCs with some modifications, and screened an antibody library for human cell surface molecules with this modified method. We succeeded in identifying CD106 (vascular cell adhesion molecule 1/VCAM1) as a potent marker to efficiently purify human cardiomyocytes derived from hESCs/hiPSCs.

## Methods

### hESC/hiPSC culture and differentiation

hESCs (KhES1) and hiPSCs (4-factor (Oct3/4, Sox2, Klf4, and c-Myc) lines: 201B6, 201B7 and 3-factor (Oct3/4, Sox2, and Klf4) lines: 253G1, 253G4) were established previously [6]–[8]. 201B6 was used as the human pluripotent cell representative in all experiments unless stated otherwise. These cells were adapted and maintained on thin-coat matrigel (Growth factor reduced; 1∶60 dilution; Invitrogen) in mouse embryonic fibroblast conditioned medium (MEF-CM) supplemented with 4 ng/mL human basic fibroblast growth factor (hbFGF; WAKO) [9]. Cells were passaged as small clumps once in every 4–6 days using CTK solution (0.1% Collagenase IV, 0.25% Trypsin, 20% Knockout serum replacement (KSR), and 1 mM CaCl_2_ in Phosphate buffered saline (PBS)) [6]. MEF cells were treated with Mitomycin-C (MMC) (WAKO) for 2.5 hours, harvested and seeded at approximately 55,000 cells/cm^2^ in MEF medium (Dulbecco's modified Eagle's medium (DMEM) containing 10% fetal calf serum (FCS), 2 mM L-glutamine, 1% nonessential amino acids (NEAA)). After 1 day, the culture medium was exchanged with ES medium (80% KNOCKOUT–DMEM, 20% KSR, 1 mM L-glutamine, 0.1 mM β-mercaptoethanol, 1% NEAA, and 4 ng/ml hbFGF; 0.5 mL/cm^2^). MEF-CM was collected daily for 7 days and supplemented with an additional 4 ng/mL of hbFGF before feeding hES/hiPS cells.

**Figure 1:**
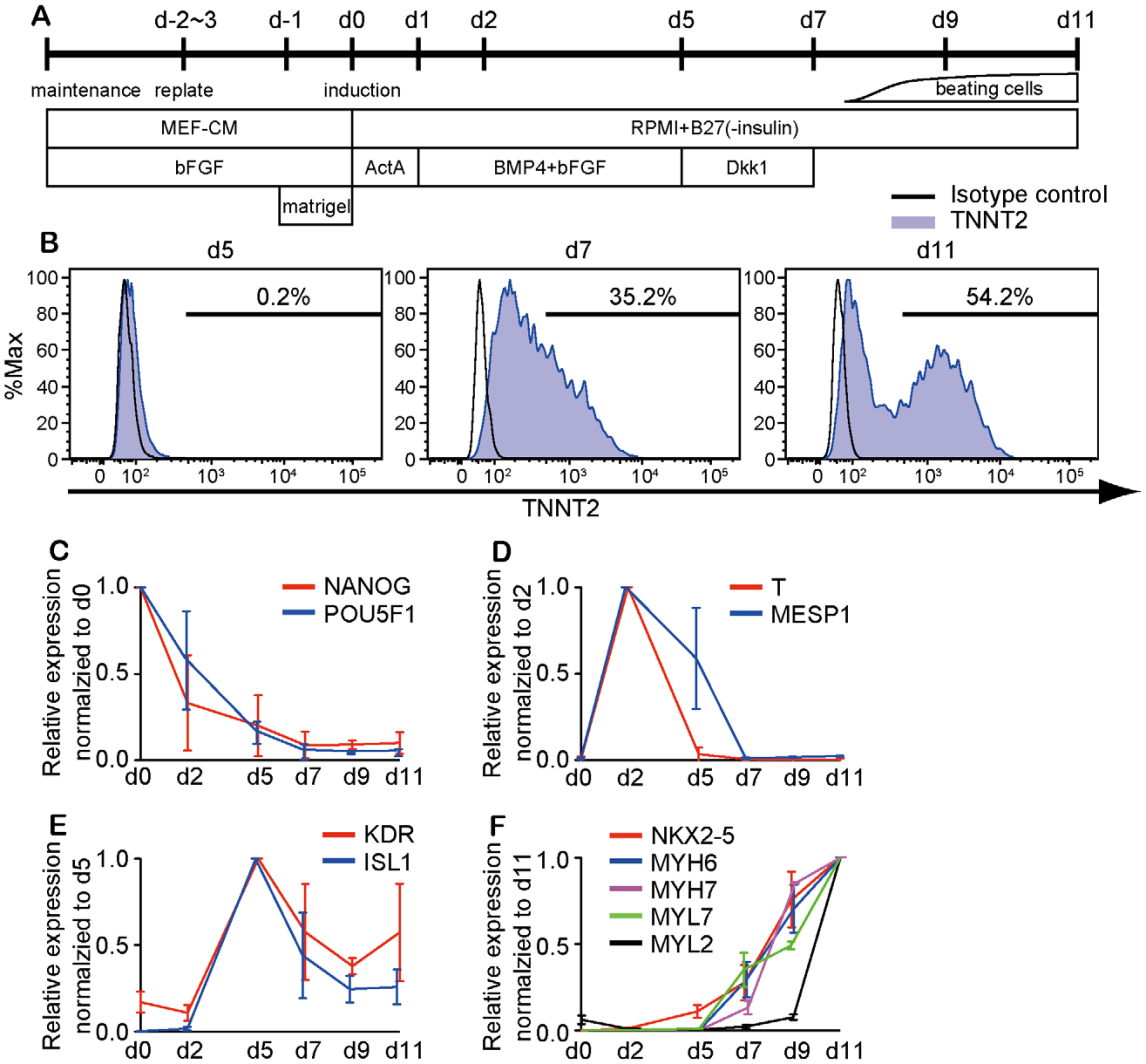
Efficient induction of cardiomyocytes from hiPSCs. (A) Schematic representation of cardiomyocyte induction protocol. (B) Expression profiles of cardiac troponin-T (TNNT2) during differentiation. Open line indicates isotype control and blue line indicates TNNT2 staining. (C–F) qPCR of differentiation stage-specific genes. (C) Pluripotency marker genes (NANOG, POU5F1); normalized to d0 expression (D) Mesodermal marker genes (T, MESP1); normalized to d2 expression (E) Cardiac progenitor genes (KDR, ISL1); normalized to d5 expression (F) Cardiac marker genes (NKX2-5, MYH6, MYH7, MYL7, MYL2); normalized to d11 expression. Mean±SD, n = 3.

Cardiomyocyte differentiation was induced as previously reported [1] with some modifications as shown in figure 1A (modified-directed differentiation protocol). Cells were detached by 3–5 min incubation with Versene (Invitrogen) and seeded onto Matrigel-coated plates at a density of 10,000 cells/cm^2^ in MEF-CM plus 4 ng/mL bFGF for 2–3 days before induction. Cells were covered with matrigel (1∶60 dilution) on the day before induction. To induce cardiac differentiation, we replaced MEF-CM with RPMI+B27 medium (RPMI1640, 2 mM L-glutamine, x1 B27 supplement without insulin) supplemented with 100 ng/mL of Activin A (ActA; R&D Systems) for 24 hours, followed by 10 ng/mL human Bone morphogenetic protein 4 (BMP4; R&D) and 10 ng/mL hbFGF for 4 days with no culture medium replacement. The culture medium was subsequently replaced with RPMI+B27 supplemented with 100 ng/mL of Dkk1 for 2 days. At day 7, the culture medium was changed to RPMI+B27 without supplementary cytokines; culture medium was refreshed every 1–2 days. Beating cells appeared at day 8–9 and beating area spread by day 11.

### Flow Cytometry, Cell Sorting, and Fluorescent Microscopy

Cells after hESC/hiPSC differentiation were dissociated by incubation with Accumax (Innovative Cell Technologies) and were stained with one of the markers listed in Table 1. For cell surface markers, staining was carried out in PBS with 5% FCS. To eliminate dead cells, cells were stained with 4′,6-diamidino-2-phenylindole (DAPI) for surface marker staining or with LIVE/DEAD fixable Aqua dead cell staining kit (Invitrogen) for intracellular staining. For intracellular proteins, staining was carried out on cells fixed with 4% paraformaldehyde (PFA) in PBS. Cells were stained with anti-cardiac isoform of Troponin T (TNNT2) (clone 13−11, Thermo Fisher scientific) labeled with Alexa-488 using Zenon technology (Invitrogen). The staining was performed in PBS with 5% FCS and 0.75% Saponin (Sigma). Stained cells were analyzed and sorted on an AriaII flow cytometer (BD). Data was collected from at least 10,000 events. For magnetic activated cell sorting (MACS; Milteny), cells were stained with anti-VCAM1 antibody conjugated with allophycocyanin (APC) followed by anti-APC microbeads (Milteny). Sorted cells were fixed and stained with TNNT2-alexa-488. Data were analyzed with DIVA software (BD) or FlowJo software (Treestar).

**Table 1:** Antibody list.

Antibody	Conjugate	Concentration	Clone	Maker
VCAM1	APC	1∶200	STA	BioLegend
PDGFRβ	PE	1∶50	28d4	BD
VE-cadherin	PE	1∶50	55-7h1	BD
TRA-1-60	FITC	1∶20	Tra-1-60	BD

APC: allophycocyanin, PE: phycoerythrin, FITC: Fluorescein isothiocianate.

For fluorescent microscopy, hiPSC-derived VCAM1-positive cells were sorted at d11 and recultured on 0.1% gelatin-coated plates in 10% fetal bovine serum (FBS)/alpha MEM (Invitrogen) for 5–7 days. Cells were stained for TNNT2, or sarcomeric α-actinin (Sigma) with DAPI.

### Surface screening of cardiomyocytes derived from hiPSCs

50,000 harvested cells were stained with each antibody from Lyoplate human cell surface marker screening panel (BD), followed by anti-Mouse IgG conjugated with Alexa-647 or anti-Rat IgG conjugated with Alexa-647. Cells stained with surface marker were fixed by 4% PFA, and stained with TNNT2-antibody labeled with Alexa-488 using zenon technology. Stained cells were analyzed on an LSRFortessa equipped with a High Throughput Sampler (BD).

### Quantitative reverse transcription polymerase chain reaction

Total RNA was prepared with the RNeasy mini kit. 500 ng RNA was reverse transcribed into cDNA via random hexamers and Oligo (dT) with Superscript III Reverse Transcriptase SuperMix (Invitrogen). Quantitative Real-time PCR (qPCR) was performed on a StepOne Plus (Applied Biosystems). All experiments were performed in biological triplicate with TaqMan fast advanced master mix (Applied Biosystems). qPCR was performed for at least three independent experiments at each time point. All TaqMan probes listed in Table 2 were purchased from Applied Biosystems. Expression level was calculated by the ddCt method and normalized to ribosomal 18S RNA and indicated time point.

**Table 2:** TaqMan probe list.

Gene symbol	Probe id
18S	Hs99999901_s1
NANOG	Hs02387400_g1
POU5F1	Hs03005111_g1
T	Hs00610080_m1
MESP1	Hs00251489_m1
KDR	Hs00911700_m1
ISL1	Hs01099687_m1
NKX2-5	Hs00231763_m1
MYH6	Hs00411908_m1
MYH7	Hs01110632_m1
MYL2	Hs00166405_m1
MYL7	Hs00221909_m1
TNNT2	Hs00165960_m1
VCAM1	Hs01003372_m1

To demonstrate transgene expression, qPCR was performed with Power SYBR Green PCR master mix (Applied Biosystems) and primers as reported previously [7]. Expression level was compared to human dermal fibroblast (HDF) 7 days after the transduction with the four retroviruses (HDF/4f-7d) [7].

### Electrophysiological studies

Electrophysiological studies were carried out as previously described [10], [11]. Briefly, VCAM1-positive cells after 11 days of induction were collected by MACS sorting and re-seeded on gelatin-coated coverslips in RPMI+B27 medium. The purified cells were cultured for 7–8 days under this condition before use. The coverslips were then transferred to a patch clamp recording chamber, and electrophysiological measurements were carried out using Axopatch200B amplifier and Digidata 1440A interface (Molecular Devices, CA). Self beating cells were selected for this study.

Composition of Solutions. Physiological bathing solution contained (in mM) 140 NaCl, 5.4 KCl, 1.8 CaCl2, 1.0 MgCl_2_, 0.33 NaH_2_PO_4_, 5 dextrose, and 10 HEPES, adjusted to pH 7.40 with NaOH. Pipette solution contained 135 KCl, 5 Na_2_ creatine phosphate, 5 MgATP and 10 HEPES, adjusted to pH 7.20 with KOH. The electrode resistance was 4–6 MOhm. The action potential recordings were carried out at 29–31°C. The temperature was measured at the beginning of each cell measurement (approximately every 30 min).

## Results

### Efficient induction of cardiomyocytes from hiPSCs

hiPSCs (201B6) were differentiated toward cardiomyocytes using modified-directed differentiation protocol (Method, Figure 1A). This differentiation method was based on a previous report [1] with the following modifications. Firstly, matrigel was added prior to induction to support cell survival during differentiation following a recent report (Zhang, Circulation, 2010: Abstract A20724). Secondly, we further added bFGF during d1-5 to enhance mesoderm differentiation and Dkk1 during d5-7 to improve cardiac differentiation from mesoderm [2], [12]. Beating clusters were first observed at day 8–9 and spread by day 11 after induction (Movie S1). TNNT2-positive cells appeared at day 7–8 after induction and were increased up to 30–70% (48±12%, n = 17) of total cells at day 11 (Figure 1B) whereas only 10% of cells from 201B6 were positive for TNNT2 without these modifications (Data not shown). qPCR analysis reflected the differentiation processes from an undifferentiated state to cardiomyocytes (Figure 1C–F). Rapid downregulation of pluripotent stem cell markers such as *NANOG* and *POU5F1* (Figure 1C) was observed within two days of differentiation. Early and cardiac mesodermal genes (*T*, *MESP1*, *KDR, ISL1*) were expressed during day 2–5 (Figure 1D and E) and cardiac genes (*NKX2-5*, *MYH6*, *MYH7*, *MYL2*, and *MYL7*) were expressed after day 7 (Figure 1F). Though very faint expressions of the *OCT3/4* and *SOX2* transgenes were detected by qPCR after day 5 (data not shown), the expression level of total Oct3/4 and Sox2 mRNA continued to be suppressed after differentiation, suggesting that reactivation of transgenes should be functionally negligible.

### VCAM1 is specifically expressed on cardiomyocytes derived from hiPSCs

**Figure 2:**
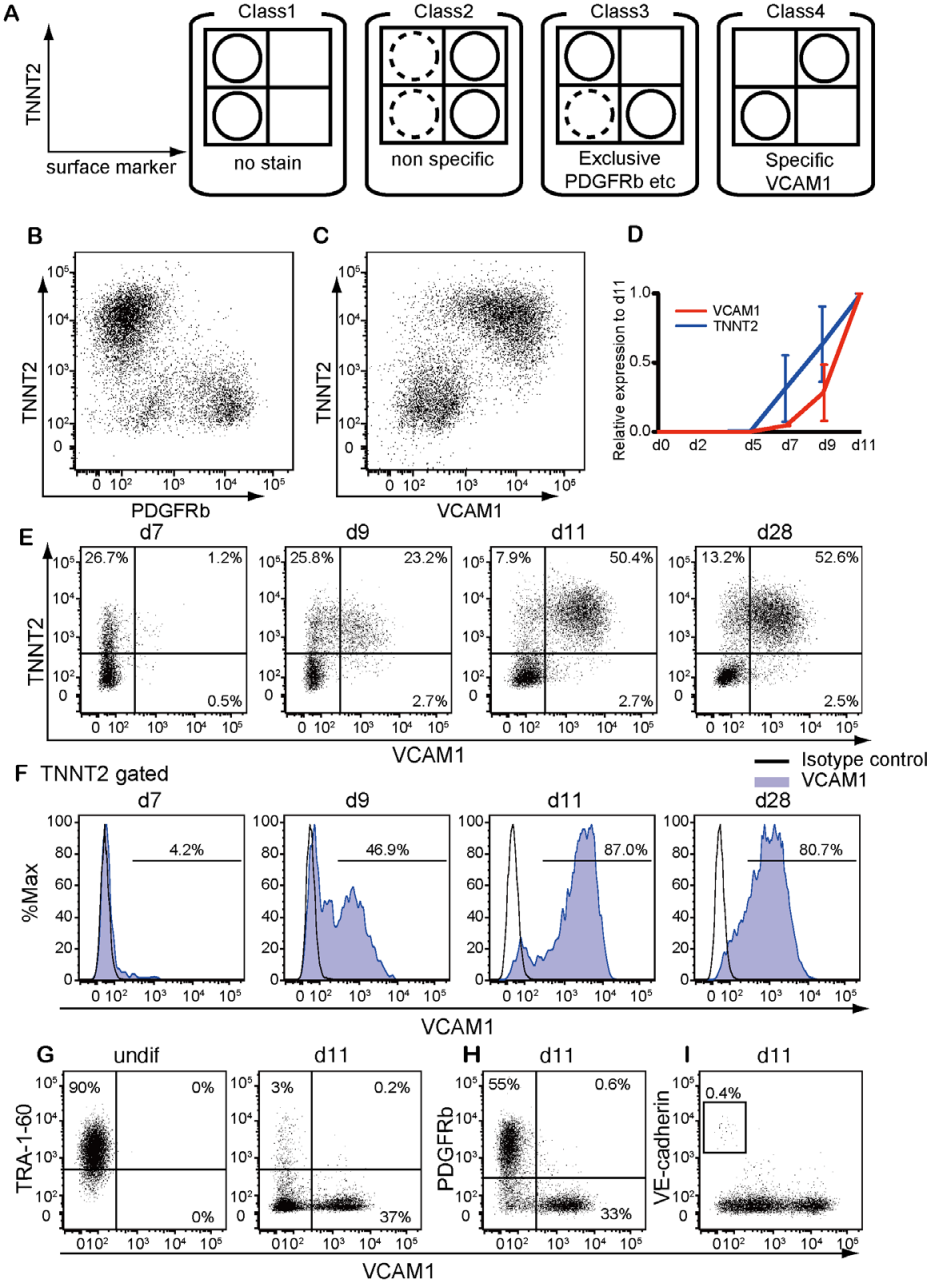
Cell surface marker screening. (A) Classification of cell surface markers and schematic dot plot diagrams for each class. (B) Exclusive expression pattern of TNNT2 and PDGFRβ (Class 3) (C) Concordant expression pattern of TNNT2 and VCAM1 (Class 4). (D) qPCR of TNNT2 and VCAM1. VCAM1 was expressed concordantly with the cardiac gene. (E–I) Representative flow-cytometry analysis. (E) Expression time course of TNNT2 and VCAM1. (F) VCAM1 expression in TNNT2-positive cells. (G–I) Dot plots of VCAM1 and TRA-1-60 (undifferentiated hESC/hiPSC marker) at day 0 and 11 (G), PDGFRβ (mesoderm/pericyte marker) (H), and VE-cadherin (endothelial marker) (I) at day 11. These markers were exclusively expressed.

Using this efficient method, we screened 242 anti-human antibodies (Lyoplate human cell surface marker screening panel (BD)) of cell surface markers on TNNT2-positive cells at day 11 after induction. We co-stained total cells at day 11 with anti-TNNT2 antibody and one of the antibodies in the library, and examined their expression pattern with flow cytometry. We classified the antibodies into four groups as class 1: no expression, class 2: non-specific, class 3: non-cardiac, and class 4: cardiac expression (Figure 2A; Dataset S1). Among them, 154 and 48 antibodies were classified as class 1 and 2. Thirty-nine antibodies including some undifferentiated ESC, endothelial, hematopoietic, and pericyte markers were classified as class 3 (Figure 2A and B). Only one antibody, anti-CD106 (vascular cell adhesion molecule 1; VCAM1) antibody was classified as class 4 (Figure 2C).

We further characterized VCAM1 as a cardiomyocyte marker. qPCR revealed that the expression of VCAM1 appeared at day 7–9 after induction (Figure 2D). Detailed expression time course of VCAM1 and TNNT2 by flow cytometry revealed TNNT2 expression preceded VCAM1 appearance (Figure 2E). A small number of TNNT2-positive cells were positive for VCAM1 at day 7 and double positive cells increased to approximately 80% of TNNT2-positive cells by day 11 (Figure 2E and F). VCAM1/TNNT-double positive cells should represent a later stage cardiomyocytes than VCAM1-negative/TNNT-positive cells.

Importantly, VCAM1 expression was mutually exclusive with an undifferentiated hESC/hiPSC marker, TRA-1-60, and a mesoderm and vascular pericyte marker, platelet-derived growth factor receptor beta (PDGFRβ/CD140b) (Figure 2G and H). Though VCAM1 was reported to be expressed on cytokine-activated endothelium, an endothelial marker, CD144 (VE-cadherin) and VCAM1 were also mutually exclusive in our study (Figure 2I). Thus, VCAM1 is an exclusive marker of cardiomyocytes appearing shortly after TNNT2.

### Purification of cardiomyocytes from hiPSC culture with VCAM1

**Figure 3:**
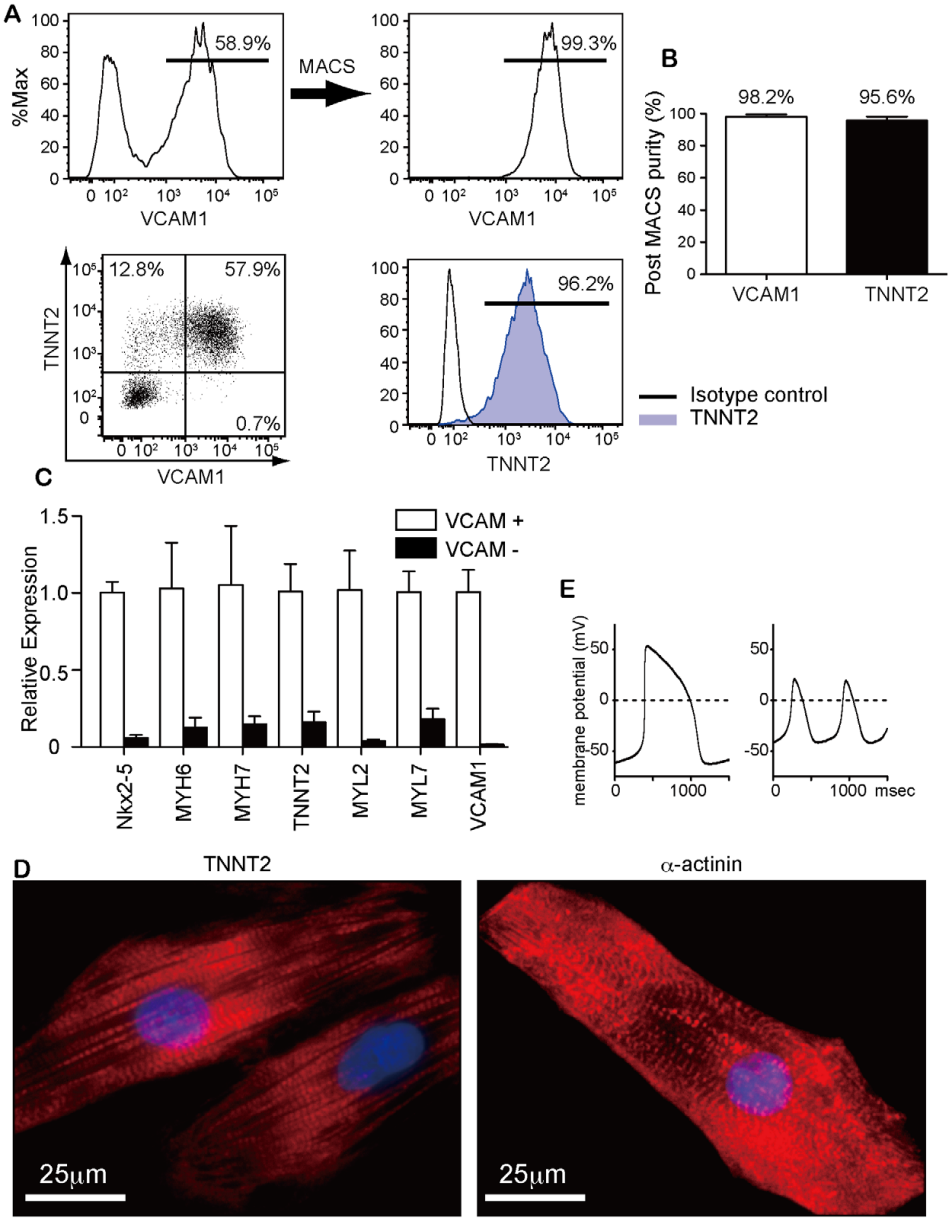
Purification of cardiomyocytes with VCAM1. (A) Representative flow-cytometry histogram of pre- and post-MACS purification with VCAM1 at d11. (B) Purity of VCAM1- and TNNT2-positive cells after MACS purification with VCAM1. 98.2±1.3% and 95.6±2.5% of sorted cells were positive for VCAM1 and TNNT2, respectively (n = 6). (C) qPCR for cardiac marker genes. Open bars: MACS-purified VCAM1-positive cells. Closed bars: control (FACS-purified VCAM1-negative cells). (D) Fluorescent staining of purified VCAM1 stained with TNNT2 or α-actinin. Clear sarcomere structures were observed. (E) Electrophysiological study of single VCAM1-positive cell. Action potentials with ventricular-like pattern (left) and pacemaker-like pattern (right).

Next, we purified VCAM1-positive cells by MACS at d11. VCAM1-positive cells were obtained by MACS with around 98% purity (98.2±1.3%, n = 6). Approximately 95% (95.6±2.5%, n = 6) of purified cells were positive for TNNT2 (Figure 3A and B). 5−10×10^5^ VCAM1-positive cells were sorted from a single well of a six-well culture plate in which 10×10^5^ undifferentiated hiPSCs were plated. Purified VCAM1-positive cells at day 11 highly expressed various mRNAs of cardiac genes compared with control (FACS-purified VCAM1-negative cells) (Figure 3C). Re-cultured VCAM1-positive cells showed structural and functional cardiomyocyte features such as clear sarcomere formation (Figure 3D), self-beating (Movie S2) and action potentials resembling ventricular and pacemaker cells (Figure 3E and Table 3). Thus, human cardiomyocytes can be highly purified with this antibody even with MACS.

**Table 3:** Summary for EPS.

Parameter	mean±SD (n = 49)
Peak (mV)	28.9±11.4
Maximum diastolic potential (mV)	−56.3±6.6
Amplitude (mV)	85.2±16.4
Action potential duration 50 (msec)	222±111
Action potential duration 30 (msec)	162±85
dV/dt (mV/msec)	33.4±9.4
Mean cycle length (sec)	1.13±0.97

### VCAM1 is a robust marker for cardiomyocytes from various human ESC and iPSC lines

**Figure 4:**
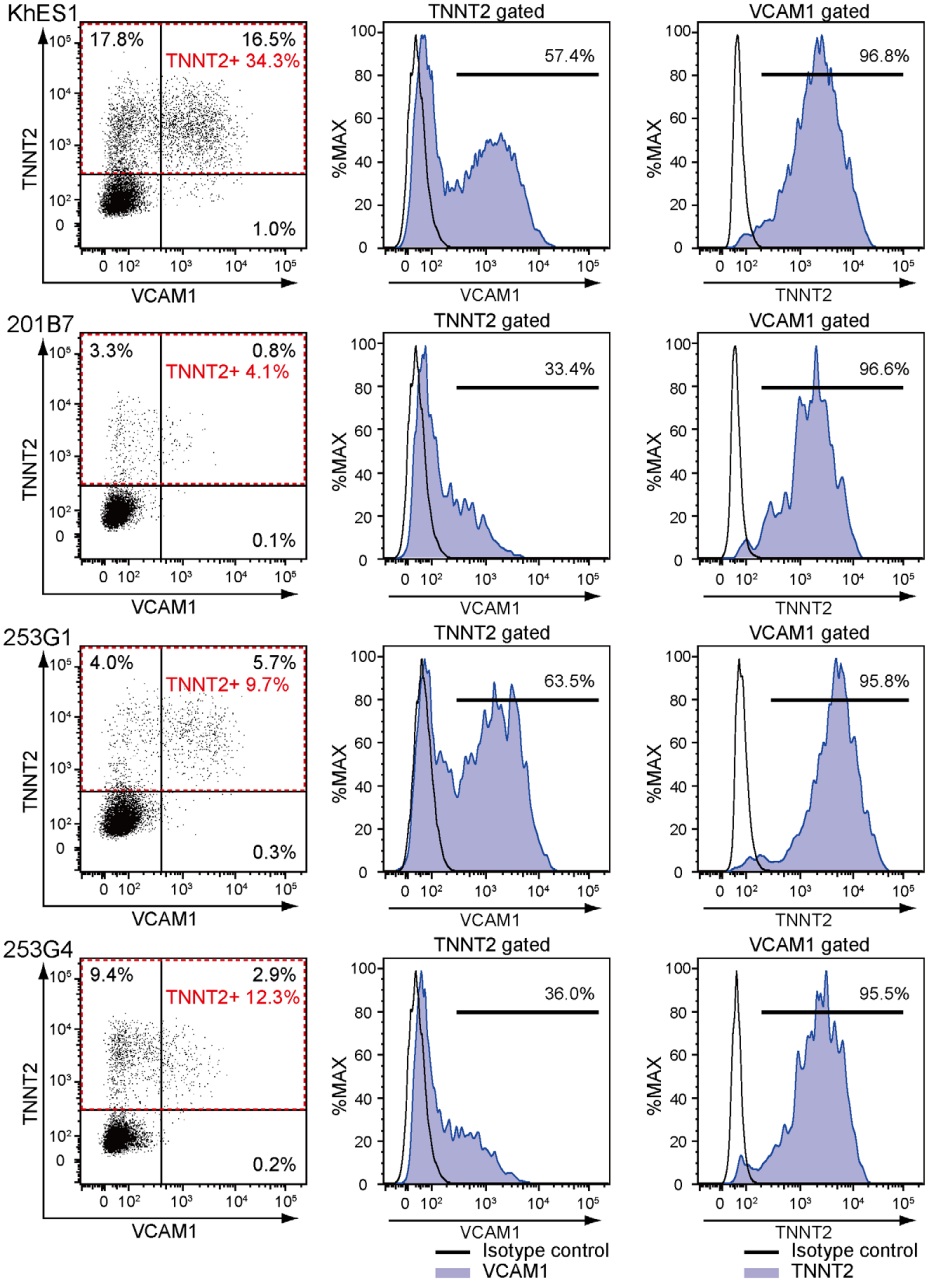
Robust expression of VCAM1 on cardiomyocytes. Representative plots of flow-cytometry for TNNT2 and VCAM1 in various hESC/hiPSC lines at day 11. Left panels: dot plots for TNNT2 and VCAM1. Middle panels: histograms for VCAM1 expression in TNNT2-positive cells. Right panels: histograms for TNNT2 expression in VCAM1-positive cells.

To further demonstrate the utility of this method, we evaluated several hESC/hiPSC lines (Figure 4). TNNT2-positive cardiomyocytes were induced from a hESC line (KhES1) and three hiPSC lines (253G1, 253G4, 201B7) with 4.1–34.3% efficiency. Differentiation efficiencies of these lines were lower than that of 201B6, but were not related to their derivation methods. VCAM1 specifically marked a subset of TNNT2-positive cells, but percentage of VCAM1-positive cells within TNNT2-positive cells varied from 33% to 64%, reflecting differences in cardiomyocyte differentiation and maybe maturation propensities among cell lines [13]. Even with such variations, TNNT2-positive cardiomyocytes could be highly purified with VCAM1 in various human pluripotent stem cell lines (>95%) (Figure 4). Thus we conclude that VCAM1 is a very useful marker to purify cardiomyocytes derived from hESCs and hiPSCs.

## Discussion

Here we reported an efficient cardiomyocyte induction method from hESCs/hiPSCs and demonstrated VCAM1 as a potent surface marker for cardiomyocytes induced with this method. Some purification methods have been previously established [14]–[16]. Tetramethylrhodamine methyl ester perchlorate, a mitochondrial fluorescent dye could selectively enrich hESC/hiPSC-derived cardiomyocytes (>99% purity) by fluorescence-activated cell sorting (FACS) [14]. As this technique depends on FACS purification, there is a scale limitation due to cell sorting capacity. ALCAM was reported as a cell surface marker for cardiomyocytes derived from a mouse embryo and hESCs [15], [17]. ALCAM is also expressed on other cell types, thus, purity using MACS was relatively low (>85%). A genetic approach, which would not be applicable for clinical purpose, was also reported using drug selection with lenti-viral vector that encodes a drug resistant gene [16]. After myocardial infarction, up to 10^9^ cardiomyocytes would be lost in human. Our method, with which we can highly purify cardiomyocytes with cell surface marker VCAM1 using MACS, is a scalable, selective and non-genetic method to prepare cardiomyocytes for clinical purpose.

With this method, a very small subset of TNNT2-negative, non-cardiomyocyte population was still observed in MACS-purified VCAM1-positive cells (<5%). Currently, the identity of VCAM1-positive/TNNT2-negative cells is still unclear. VCAM1 was negative for undifferentiated hESCs/hiPSCs, a mesoderm and vascular pericyte marker, PDGFRβ/CD140b, and an endothelial marker, CD144 (VE-cadherin) (Figure 2G–I). VCAM1 expression is reported in developing allantois and the heart [18], and bone marrow stroma [19]. Thus, VCAM1-positive/TNNT2-negative cells should probably be mesoderm derivatives or mesenchymal lineage cells, but should not include undifferentiated cells.

To apply this method for clinical application, transplantation methods should be developed. Regenerative potential and side effects such as tumor formation should be examined in each transplantation method. There was almost no undifferentiated cell contamination after purification, but safety issue in our method should be confirmed before moving to clinical usage. Nevertheless, this efficient cardiomyocyte induction method and a potent cell surface marker for robust, efficient and scalable purification of cardiomyocytes from hESCs/hiPSCs would offer a valuable technological basis for future cardiac cell therapy using hESCs/hiPSCs.

## Supporting Information

Dataset S1 **Results of cell surface marker screening.** Percentages of each quadrant, classification and antibody information for each antibody are shown.
(XLS)

Movie S1 **Beating clusters at day 11 after induction.**
(MP4)

Movie S2 **Single beating cell at day 5 after VCAM1 sorting.**
(MP4)
